# Quantitative Markers of Neural Changes, Retinal Thickness, and Responses to Electrical Stimulation in Retinal Degeneration

**DOI:** 10.1016/j.xops.2026.101174

**Published:** 2026-03-27

**Authors:** Hallur Reynisson, Maximillian Nguyen, Michael Kalloniatis, Lisa Nivison-Smith, Mohit N. Shivdasani

**Affiliations:** 1 School of Biomedical Engineering, University of New South Wales, Sydney, Australia; 2 School of Optometry and Vision Science, University of New South Wales, Sydney, Australia; 3 Neuroscience Research Australia, Sydney, Australia; 4 University of Houston College of Optometry, Houston, Texas; 5 Tyree Institute of Health Engineering (IHealthE), Sydney, Australia

**Keywords:** Photoreceptor degeneration, Retinal remodeling, Neurodegeneration, Retinal prosthesis, Electrical stimulation

## Abstract

**Purpose:**

Inherited retinal dystrophies demonstrate significant heterogeneity in the severity and distribution of photoreceptor loss. Current quantitative methodologies, however, do not account for this within-eye variation, which may explain conflicting findings regarding the efficacy of interventional strategies.

**Design:**

We developed an eccentricity-agnostic measure of photoreceptor loss and determined if accounting for localized changes in retinal degeneration could explain the performance of an implanted retinal prosthesis.

**Subjects:**

Hematoxylin and eosin stained, full thickness retinal cross sections of control eyes and eyes of an adenosine triphosphate-induced feline model of retinal degeneration (n = 4) were imaged.

**Methods:**

Individual regions of 250 μm length were quantified for localized photoreceptor degeneration using the Outer Retina ratio (ORr). Regions were categorized into no, mild, moderate, or severe degeneration groups.

**Main Outcome Measures:**

Outer Retina ratio was correlated to neural markers using 3,3′-diaminobenzidine or fluorescent immunohistochemistry to determine if localized remodeling occurred within ORr defined regions. Outer Retina ratio was also assessed against measures of cortical neural activity evoked by electrical stimulation through a suprachoroidally implanted electrode array.

**Results:**

Outer Retina ratio effectively correlated with changes in rhodopsin and cone opsin labeling in the outer retina as well as inner retinal changes in calbindin, protein kinase C alpha, and RNA-binding protein with multiple splicing labeling. Assessment of degenerative retina categorized using ORr also identified disease alterations missed via pooled analyses including the presence of reduced opsin immunoreactivity and retraction in regions yet to exhibit signs of photoreceptor loss. There were some differences in cortical measures between ORr groups and weak individual correlations (*r* = −0.1 to 0.24; *P* < 0.05) between cortical and retinal remodeling measures, however, no single retinal remodeling metric stood out as a predominant predictor of stimulation performance.

**Conclusions:**

These findings suggest that ORr is a valid estimator of heterogeneous degeneration in a retinal degeneration model and could be useful to identify areas with specific degenerative features in retinal degeneration. However, more work is needed to determine the quantitative remodeling metrics (and other nonretinal multi-factors) that best serve as estimates for the efficacy of therapeutic interventions.

**Financial Disclosure(s):**

Proprietary or commercial disclosure may be found in the Footnotes and Disclosures at the end of this article.

Photoreceptor degenerations such as retinitis pigmentosa and age-related macular degeneration are leading causes of irreversible vision loss and the target of several vision-restoration intervention strategies, including gene therapy, stem cell transplantation, and electrical stimulation via retinal prostheses.^1^ A major challenge in developing such interventions, however, is the heterogeneous nature of photoreceptor loss in these diseases, which shows significant variation between eyes of the same disease stage and between different retinal locations within a single eye.^2^ This variation leads to subsequent differences in the spatial and temporal distribution of secondary remodeling and neurodegenerative processes, which can have specific effects on the efficacy of interventions.^3^

Accounting for photoreceptor degeneration heterogeneity in intervention design and testing is hindered by the lack of robust, validated quantitative methodology. For example, although many pharmacologically induced models of photoreceptor degeneration using different agents (i.e., sodium iodate, iodoacetic acid, N-methyl-N-nitrosourea, and adenosine triphosphate [ATP]) and species (rodent, feline, lagomorph, ground squirrel, and swine) have reported intereye and intraeye variations in photoreceptor loss, most accounts are based on qualitative evidence (images of photoreceptor loss varying in regions adjacent to one another).^4^, ^5^, ^6^, ^7^, ^8^, ^9^, ^10^ Some studies have used quantitative methods including Tao et al^11^ who quantified photoreceptor loss in the N-methyl-N-nitrosourea induced mouse model of degeneration and found that specific retinal quadrants had more rapid photoreceptor loss than others, due to asymmetric upregulation of apoptotic markers. We also demonstrated that machine learning models could identify significant heterogeneity in a feline ATP model that correlated with independent expert analyses when assessing the severity of degeneration.^12^ However, these methods have so far had a limited scope and do not address issues such as normal species-specific variations in retinal architecture and circuitry or covariance with eccentricity.

The aim of this study was therefore to validate an eccentricity-agnostic methodology that quantifies focal photoreceptor degeneration and other subsequent retinal remodeling feature variance in a pharmacologically induced model of retinal degeneration. We also examined whether this eccentricity-agnostic method provides additional context for the interpretation of outputs of a disease intervention in this model. Specifically, we used the feline ATP model of retinal degeneration, which exhibits rapid photoreceptor loss via binding of ATP to the purinergic receptor P2X_7_ of photoreceptors, leading to calcium influx, caspase activation, and ultimately photoreceptor apoptosis.^8^, ^9^, ^10^^,^^13^, ^14^, ^15^ The ATP model demonstrates nonuniform photoreceptor degeneration similar to human disease^5^^,^^10^^,^^15^ and heterogeneity in morphological changes of bipolar, horizontal, amacrine, ganglion, and Müller cells.^13^^,^^15^ The feline ATP model has also been the subject of interventions involving electrical stimulation via a microelectrode array implant and some correlation between the level of retinal glial activity and thresholds for cortical activity evoked by retinal electrical stimulation have been shown.^16^ This study further assessed photoreceptor degeneration in the feline model, accounting for variations with eccentricity, and determined if this can explain localized variations in inner retinal neuron morphology and subsequently, performance of stimulation of individual electrodes of a retinal prosthesis.

## Methods

### Animals

**Figure 1:**
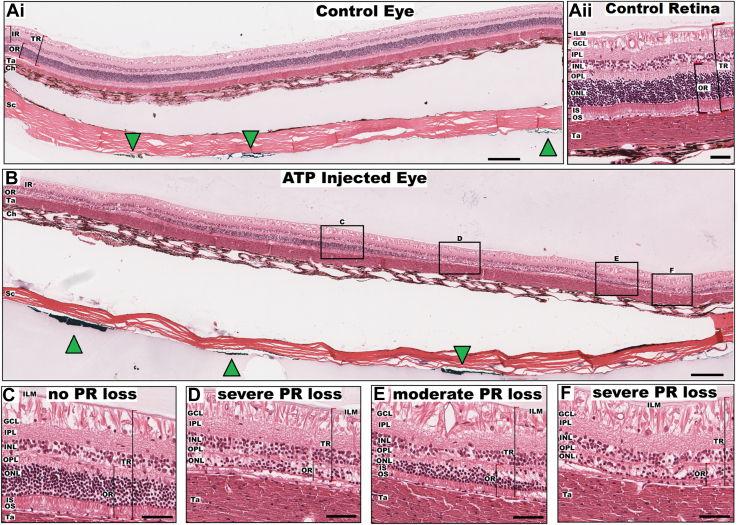
Examples of control and adenosine triphosphate (ATP) treated retina analyzed for Outer Retina ratio (ORr). Retina of a healthy control eye, in low (**Ai**) and high (**Aii**) magnification, showing uniform thickness of the outer and IR as a function of eccentricity. The pocket between the Ch and the Sc indicates placement of the stimulating microelectrode array (green arrowheads indicate electrode locations). **B,** Retina of an ATP-treated eye within the implanted region with variable thickness and ONL loss. Black boxes indicate regions of 250 μm in length used for ORr analysis. Magnification views demonstrate heterogeneous regions of (**C**) no PR loss (ORr_C_ = 0.46 ± 0.02) adjacent to regions of (**D**) severe PR loss (ORr_D_ = 0.20 ± 0.02). Other regions displayed (**E**) moderate PR loss (ORr_E_ = 0.31 ± 0.03) flanked by areas of (**D, F**) severe PR loss (ORr_F_ = 0.19 ± 0.01). Scale bar for **Ai** and **B** is 250 μm, and 50 μm for **Aii** and **C–F**. Ch = choroid; GCL = ganglion cell layer; ILM = inner limiting membrane; INL = inner nuclear layer; IPL = inner plexiform layer; IR = inner retina; IS = inner segments; ONL = outer nuclear layer; OPL = outer plexiform layer; OR = outer retina; OS = outer segments; PR = photoreceptor; Sc = sclera; Ta = tapetum; TR = total retina.

All studies were approved by the Bionics Institute Animal Research Ethics Committee under project #14 304AB, in accordance with the Australian Code for the Care and Use of Animals, the Association for Research in Vision and Ophthalmology statement, and National Institutes of Health guidelines. This study used paraffin embedded retinae obtained from 4 felines (n = 8 eyes) from our previous reported studies.^17^^,^^18^ Briefly, normally sighted feline eyes were unilaterally injected intravitreally with 200 μL of 0.2M ATP under general anesthesia.^17^^,^^18^ The ATP salt was dissolved in 0.9% saline with 4 mg/mL dexamethasone added to reduce inflammation. Clinical assessment of vision loss was assessed in the injected eye by analysis of the scotopic a-wave amplitudes during full-field flash electroretinogram (Espion; Diagnosys LLC), while the animals were under general anesthesia as reported by Halupka et al.^17^ Stimulus intensities ranged from 0.001 to 10 cd.s/m^2^ post a 20-minute dark adaptation period. Adenosine triphosphate injected eyes were considered to have achieved vision loss when the electroretinogram scotopic a-wave had diminished by ≥50% in amplitude. Twelve to 23 weeks postinjection, all animals were subject to a terminal electrophysiology experiment, where a platinum microelectrode array (8 × 17 mm) was implanted into the suprachoroidal space and electrical stimulation performance was assessed using measures of neural spiking activity evoked in the visual cortex.^17^^,^^18^ Animals were then euthanized, transcardially perfused, and both eyes enucleated. Retinae were dissected, fixed, and paraffin embedded for sectioning. Individual electrode locations were marked using fluorescent dyes at the time of dissection (Fig 1A, B, green arrows). Groups of consecutive (5 μm interval) sections were cut serially from close to the optic nerve to the temporal edge of the array and retained. Some tissues were stained with hematoxylin and eosin (H&E) and analyzed in previous studies^12^^,^^19^^,^^20^ (Fig 1A–F), whereas other tissues were further stained and used in this study.

### Immunostaining and Imaging

For immunofluorescent labeling, paraffin sections were deparaffinized in xylene and rehydrated with graded ethanol to 0.1M phosphate buffer, pH 7.4. Tissue then underwent antigen retrieval in citrate buffer (0.01% [w/v] citrate, 0.5% [v/v] Triton-X, pH 6.0) at 70 to 75 °C for 15 minutes. Slides were cooled to room temperature, blocked with 10% (v/v) goat serum and 0.1% (v/v) Triton-X in 0.1M phosphate buffer for 1 hour, and then incubated overnight at 4 °C with primary antibodies for fluorescent immunohistochemistry labeling (Table 1). Tissue was washed in 0.1M phosphate buffer and incubated with secondary antibodies: mouse Alexafluor 488 and rabbit Alexafluor 594 (1:500 dilution; Invitrogen), for 2 hours at room temperature. Tissue was counterstained with 4′,6-diamidino-2-phenylindole (DAPI; 1:1000 dilution; Sigma-Aldrich).

**Table 1:** Details of the Antibodies Used in This Study

Antigen	Immunogen	Specificity∗	Manufacturer, Catalog No.	Host	Dilution	Retinal Cell Types Labeled
Rhodopsin	Recombinant bovine rhodopsin	Binds to the rhodopsin (N terminal)	Abcam, ab98887	Ms; monoclonal	1:1000	Rhodopsin found in rod outer segments
Red/green cone opsin	Recombinant human red/green opsin	This antibody detects red-sensitive opsin/green-sensitive opsins	Millipore, AB5405	Rb; polyclonal	1:100	Opsins found in red/green cone outer segments
Blue cone opsin	Recombinant human blue opsin	Recognizes opsin, blue	Millipore, AB5407	Rb; polyclonal	1:100	Opsins found in blue cone outer segments
Calbindin	Purified bovine kidney calbindin-D-28K	The antibody binds to calcium-binding proteins	Sigma-Aldrich, C9848	Ms; monoclonal	1:1000	Subpopulations of amacrine cells and horizontal cells
Calretinin (CR)	Rat calretinin, amino acids 38–151	The antibody binds to the Ca^2+^ binding protein calretinin	BD Transduction, 610908	Ms; monoclonal	1:1000	Populations of amacrine and ganglion cells
Protein kinase C-α (PKCα)	Purified bovine brain PKC	Reacts with the 80 kDa polypeptide of PKC	Sigma-Aldrich, P5704	Ms; monoclonal	1:400	Rod bipolar cells

Ms = mouse; Rb = rabbit.

∗ Specificity from manufacturers' websites.

For 3,3′-diaminobenzidine (DAB) immunohistochemistry labeling, the Bond Chromoplex 1 dual detection kit was used according to the manufacturer’s instructions (DS9477; Leica Biosystems). Briefly, paraffin embedded sections were dewaxed and ethylenediaminetetraacetic acid antigen retrieval performed. Sections were then blocked with 4% hydrogen peroxide after incubation with primary antibodies (Table 1) for 60 minutes at room temperature. Detection of mouse and rabbit primary antibodies was performed using antimouse poly-horseradish peroxidase and antirabbit poly-alkaline phosphatase in 10% (v/v) animal serum in Tris-buffered saline and 0.09% ProClin 950 with DAB and Red substrate. Sections were counterstained with hematoxylin then dehydrated in 100% ethanol followed by xylene before coverslipping. All imaging was carried out on the Vectra Polaris slide scanner (PerkinElmer).

### Image Processing

Microscope images of the H&E-stained sections were segmented into adjacent regions of 250 μm length, within which various features were quantified, from both H&E and corresponding immunohistochemistry images belonging to the same group. Regions in H&E images were inspected sequentially to identify those with the presence of a suprachoroidal pocket indicating that the region was in contact with the multielectrode array (Fig 1A, B). Those containing a suprachoroidal pocket were further inspected for dye markings to determine local regions directly above stimulating electrodes and the electrode number corresponding to each dye marking was noted for later correlation analysis with the electrophysiology data. Immunolabeling was assessed using ImageJ (version 1.53n, National Institutes of Health), custom MATLAB scripts using the Image Processing Toolbox (R2020b, v9.9.0, Mathworks), and custom Python scripts (v3.10.5) using the NumPy,^21^ SciPy,^22^ scikit-posthocs, pingouin, and scikit-learn packages.

### Outer Retina Ratio

For all segmented regions, localized photoreceptor loss was determined from the H&E images as the Outer Retina ratio (ORr): the ratio of outer retina (OR) thickness, including the outer segments, inner segments, outer nuclear layer (ONL), and outer plexiform layer (OPL); to the total retina thickness including the OR, inner nuclear layer (INL), inner plexiform layer (IPL), ganglion cell layer (GCL), and inner limiting membrane (Fig 1). Outer Retina ratio values were determined at 50 μm intervals for each 250 μm long H&E-stained retinal image, then averaged to get a mean ORr per region. Each 250 μm region neighbored the next, such that regions were chosen without any bias. Calculating a ratio rather than absolute thicknesses meant that the plane of the retinal region did not have to align perfectly in the vertical dimension, and any minor obliqueness in the section angle due to oblique cutting would be accounted for. This was done particularly as we were evaluating entire-retina sections where obtaining perfectly vertical sections can be challenging. Such normalization for other analyses has been previously done where immunohistochemistry labeling was analyzed as a function of the depth of the IPL.^23^

**Figure 2:**
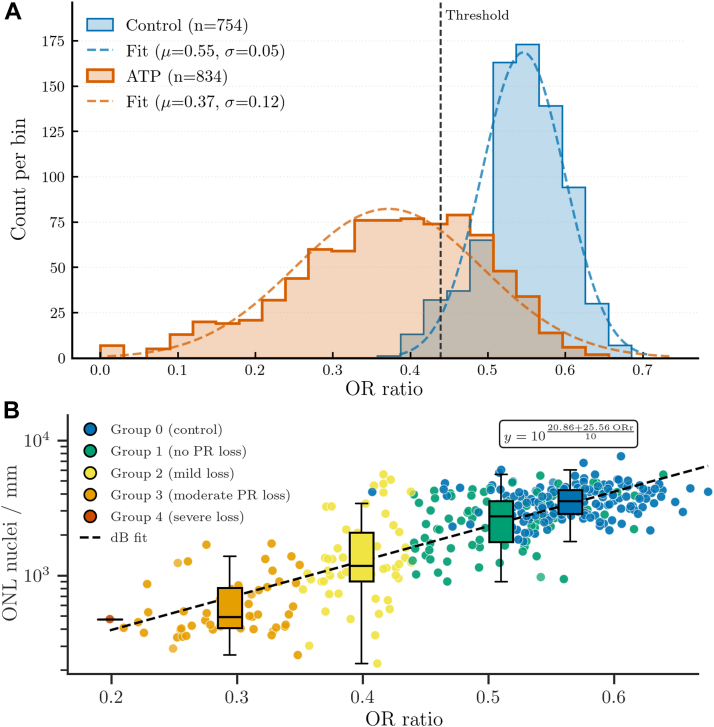
Distribution of Outer Retina ratio (ORr) and its relationship to outer nuclear layer (ONL) nuclei. (**A**) Distribution of ORr of control eyes (blue) and adenosine triphosphate (ATP) treated eyes (orange) in frequency bins (left y-axis) and their respective fitted normal distributions (dashed colored lines). The cutoff value (dashed black line) used to separate ATP group 1 (no obvious degeneration) from groups 2 (mild degeneration), 3 (moderate degeneration), and 4 (severe degeneration) is based on the mean and standard deviation of the ORr of the control. (**B**) Relationship between ORr and cell counts in the ONL per millimeter shown as individual scatter points and quartile box plots. Note, with group 4, for all images except one data point, an ONL nuclei count could not be determined because of severe degeneration, obscuring delineation of the ONL with other layers. A log scale fit is shown with a black dashed line with the fitted equation in the top right. OR = outer retina; PR = photoreceptor.

A threshold value for normative ORr was calculated as 2 standard deviations below the mean ORr obtained from all images from the control eyes (Fig 2A, 0.44 in this study). All images of ATP-treated eyes were then assigned to degeneration groups based on their mean ORr relative to the threshold value. Specifically, images of ATP-treated eyes were classified into group 1 (no degeneration) if the mean ORr was equal or greater than the normative threshold value; group 2 (mild degeneration) if the mean ORr was less than the normative threshold value, but ≥0.35; group 3 (moderate degeneration) if the mean ORr was <0.35 but ≥0.2; or group 4 (severe degeneration) if the mean ORr was <0.2. All control eye images were assigned to group 0 regardless of ORr value. Therefore, we set group 0 as control eyes regardless of ORr, in contrast with group 1, which consisted entirely of ATP-injected eyes that had statistically the same ORr values as the controls. The further categorization of ORr into groups 2 to 4 was for categorical analysis of the continuous variable ORr between groups 0 and 4, though the cutoff points between groups 2 and 3 (ORr = 0.35) and groups 3 and 4 (ORr = 0.2) were arbitrarily picked.

### Quantification of Photoreceptor Cell Markers

Assessment of photoreceptor nuclei and rhodopsin and cone opsin markers were only performed for images of regions outside of the suprachoroidal pocket to minimize confounding damage from implantation, as correlation to cortical metrics from electrical stimulation was only conducted with inner retinal markers. In addition, photoreceptor markers were not analyzed in images assigned to group 4 because of the severity of degeneration and inability to reliably delineate the outer retinal layers. Rhodopsin immunoreactivity in the outer segments was measured using the ImageJ magic wand tool, subtracted from background (where background is based on mean rhodopsin immunoreactivity of the choroid), and normalized against the mean rhodopsin immunoreactivity of all images of the control eye for that respective animal. Red/green/blue cone opsins were quantified together via the same method. Nuclei counts within the ONL were assessed using the ImageJ “analyze particles” function (Size = 0 – infinity pixel units, circularity = 0.00–1.00) and the watershed function on DAPI labeling nuclei. Rhodopsin/cone opsin positive nuclei in the ONL were manually counted.

### Quantification of Inner Retinal Neuron Markers

Assessment of inner retinal neuron nuclei and cell markers were performed for all segmented regions but later correlation analyses with cortical measures were only performed with identified regions directly above stimulating electrodes. 4′,6-Diamidino-2-phenylindole-labeled nuclei of the INL and GCL were manually counted. Labeling of inner retinal neurons by calbindin, calretinin, or protein kinase C alpha (PKCα) was quantified by identifying positive DAB pixels based on the red, green, and blue channels chromatic components of images. Threshold criteria were derived to ensure differentiation of brown hues from neighboring color spectra in the stained images. The total area of positive DAB labeled pixels was then calculated and normalized by dividing by the length of retina analyzed.

### Retinal Marker and Cortical Activity Correlation

Cortical activity was analyzed in response to monopolar stimulation of individual electrodes on the implanted array described in Spencer et al.^18^ In brief, the stimulation protocol consisted of symmetric biphasic pulses applied to each retinal electrode compared with an extraocular platinum return electrode, using a 1000 μs phase width, 25 μs interphase gap, 0 to 750 μA amplitude range, and 1 Hz frequency. Neural activity was recorded in both hemispheres using multichannel recording arrays (Blackrock Microsystems). Thresholds for each recording channel were defined as the injected current required to elicit 50% of the maximum number of spikes in a window 3 to 20 ms poststimulation. For each stimulating electrode, the lowest cortical thresholds of evoked neural activity obtained from the ipsilateral and contralateral hemispheres, as well as a d-prime (d′) cortical selectivity, defined as the rate of reduction in normalized firing across the cortical array as a function of distance from the best cortical channel (one that had the lowest threshold)^18^^,^^24^, ^25^, ^26^ were determined. Cortical activity measurements were correlated to ORr and individual inner retinal marker immunoreactivity using Pearson and Spearman correlations, to provide a comprehensive analysis of both linear and monotonic relationships, respectively.

### Statistical Analysis

All variables are expressed as mean ± standard error. Statistical analyses were conducted using Kruskal–Wallis tests followed by Holm–Bonferroni adjusted Dunn post hoc tests for multiple comparisons between ORr groups. Pearson and Spearman correlations were performed to correlate retinal findings to cortical metrics. Analyses were performed using Python (v3.10.5, Python Software Foundation), with SciPy,^22^ scikit-posthocs, pingouin, and scikit-learn packages.

## Results

### Characterizing Photoreceptor Loss in the ATP-Treated Retinae Using the ORr

Figure 2A demonstrates the ORr distribution of all control (n = 754) and ATP-treated retinal images (n = 834). Mean ORr of ATP-treated eyes was significantly reduced relative to the control group (ORr_control_: 0.55 ± 0.05, ORr_ATP_: 0.37 ± 0.12; *P* < 0.0001; Dunn test following Kruskal–Wallis). The distribution of ORr also significantly differed between groups, with control retinae showing a narrower range of ORr values (0.39–0.69), whereas ATP-treated retinae demonstrated a wider ORr range (0.00–0.64). An ORr value of 0.44 was set as a normative threshold value for retinae with no photoreceptor loss based on being equivalent to 2 standard deviations below the mean ORr of the control group (Fig 2A, dotted line). This value corresponds to the 2.3rd percentile of the control data. Subsequently, all images from control eyes were grouped into group 0 and n = 272 images of ATP-treated retinae with an ORr above 0.44 were categorized as group 1—ATP-treated retinae that did not differ from control eyes in terms of ORr. Remaining ATP-treated retinal images were assigned to group 2—mild photoreceptor degeneration (ORr, 0.35–0.44; n = 220), group 3—moderate photoreceptor degeneration (ORr, 0.2–0.35; n = 263), and group 4—severe photoreceptor degeneration (ORr < 0.2; n = 79). The distribution of groups 1 to 4 within each ATP-treated eye varied, highlighting that ORr could reflect variation both within a single eye and between different eyes (Fig S3, available at www.ophthalmologyscience.org). Outer Retina ratio defined groups also showed a positive relationship with a traditional measure of photoreceptor loss, that is, ONL nuclei count (Fig 2B), although subsequently, we quantitatively analyzed this relationship with photoreceptor markers. Modeling the ONL cell density (${CD}_{ONL}$; cells per millimeter) in log linear space as a function of ORr using ordinary least squares by fitting the data to the equation $10\;\log_{10}{CD}_{ONL}=A+B\times ORr$ resulted in the back-transformed mean fit ${CD}_{ONL}=10^{\left(\frac{20.86+25.56\times ORr}{10}\right)}$ (see Fig 2B, black dashed line). Goodness of fit assessed in the transformed space gave $R_{dB}^{2}=0.656$, ${RMSE}_{dB}=1.96dB$; multiplicative root mean square error factor F=1.57×, and the Duan smearing factor S = 1.110.

### Photoreceptor Morphology Relative to ORr

**Figure 4:**
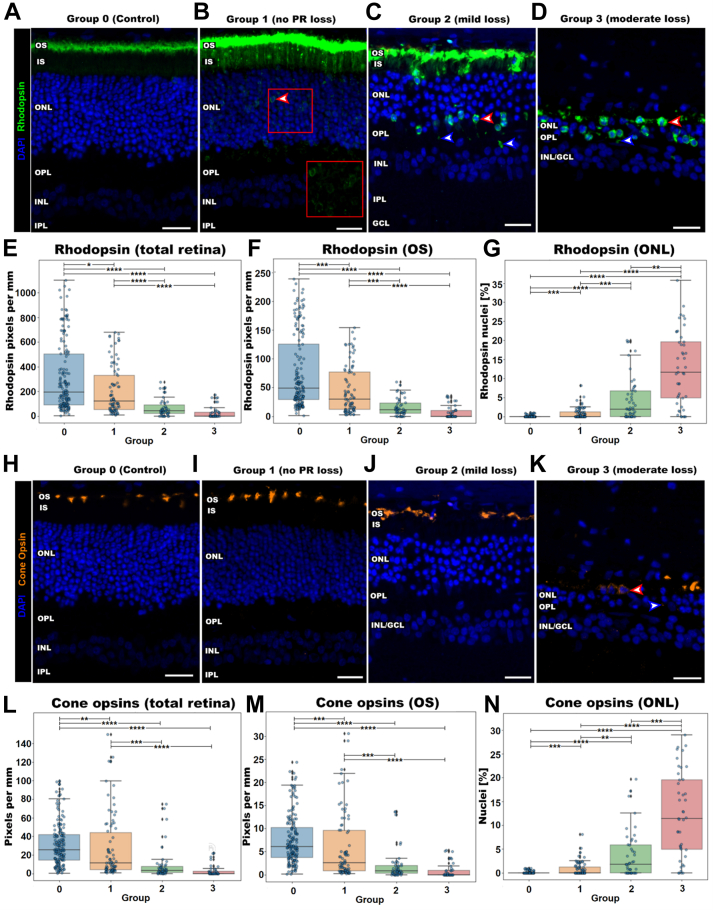
Photoreceptor opsin labeling relative to Outer Retina ratio. Representative images of rhodopsin (green) and 4′,6-diamidino-2-phenylindole (DAPI; blue) immunolabeling of (**A**) group 0 (**B**) group 1, (**C**) group 2, and (**D**) group 3. Red arrows indicate rhodopsin positive nuclei in the ONL and blue arrows indicate aberrant rhodopsin immunoreactivity in the OPL. Red box in part (**B**) has DAPI removed for clearer visualization of rhodopsin in the ONL. Quantification of rhodopsin immunoreactivity in the (**E**) total retina, (**F**) outer segment layer, and (**G**) rhodopsin positive nuclei in the ONL. Representative images of cone opsins (red) and DAPI (blue) in parts (**H**) group 0 (control), (**I**) group 1, (**J**) group 2, and (**K**) group 3. Red arrows indicate cone opsin in the ONL. Quantification of cone opsin immunoreactivity in parts (**L**) the total retina, (**M**) outer segment layer, and (**N**) cone opsin positive nuclei in the ONL. Scale bars are 50 μm. GCL = ganglion cell layer; INL = inner nuclear layer; IPL = inner plexiform layer; IR = inner retina; IS = inner segments; ONL = outer nuclear layer; OPL = outer plexiform layer; OS = outer segments; PR = photoreceptor. ∗*P* < 0.05; ∗∗*P* < 0.01; ∗∗∗*P* < 0.001; ∗∗∗∗*P* < 0.0001.

We next assessed opsin immunoreactivity and distribution within ORr defined groups to determine if ORr could accurately describe localized variations in photoreceptor morphology. Quantification was performed on all groups except group 4 because of the inability to reliably delineate the ONL and confirm opsin localization. Representative images of rhodopsin immunoreactivity from each ORr group demonstrate decreased outer segment labeling and increased aberrant ONL and OPL labeling with increasing ORr group severity (Fig 4A–D, red and blue arrows). This was supported by quantification, which showed a significant dose-dependent decrease in rhodopsin immunoreactivity within the total retina and outer segments from groups 0 to 3 (*P* <0.05–0.0001; Dunn test following Kruskal–Wallis; Fig 4E, F) and significant dose-dependent increase in rhodopsin positive nuclei in the ONL (*P* < 0.01–0.0001; Dunn test following Kruskal–Wallis; Fig 4G).

Cone opsin immunoreactivity (combined red/green and blue) revealed similar findings to rhodopsin (Fig 4H–K). Cone opsin immunoreactivity of the total retina and the outer segment layer was significantly reduced between group 0 (control) and groups 2 and 3 (mild and moderate degeneration; *P* < 0.01–0.0001; Dunn test following Kruskal–Wallis) but not between group 0 and group 1 (Fig 4L, M). Cone opsin positive nuclei in the ONL showed a dose-dependent increase across all ORr groups (*P* < 0.01–0.0001; Dunn test following Kruskal–Wallis; Fig 4N). Together, these results suggest a strong relationship with the ORr group and existing markers of retinal degeneration: canonical opsin loss and opsin retraction into the ONL.

### Inner Retinal Neuron Morphology Relative to ORr Groups

**Figure 5:**
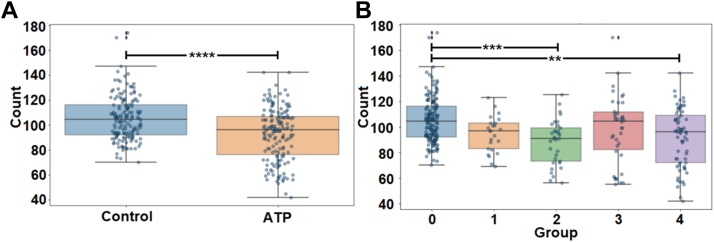
Inner nuclear layer nuclei count in relation to Outer Retina ratio (ORr) groups. Comparison of (**A**) control (group 0, n_control_ = 155) to all adenosine triphosphate (ATP) treated retinae (groups 1–4 combined, n_ATP_ = 160) and (**B**) ORr group 0 (n = 155) group 1 (n = 24), group 2 (n = 33), group 3 (n = 38), and group 4 (n = 65). ∗∗∗*P* < 0.001; ∗∗∗∗*P* < 0.0001.

Because ORr could segregate heterogeneous degenerative changes of the OR, we next assessed if ORr could be used for the same purpose for the inner retina. First, we assessed ORr relative to total INL nuclei count (Fig 5A). We found that the mean INL nuclei count was significantly lower in all ATP-treated retinal images versus control (Nuclei_control_ = 105.54 ± 1.45; Nuclei_ATP_ = 92.94 ± 1.73; *P* <0.0001; *t* test; Fig 5A). However, for ORr group comparisons, we found that INL nuclei count was only was significantly different between control (group 0) and ATP-treated group 2 and group 4 (*P* < 0.01–0.001; Dunn test following Kruskal–Wallis) with no significant difference between ATP-treated groups (Fig 5B).

**Figure 6:**
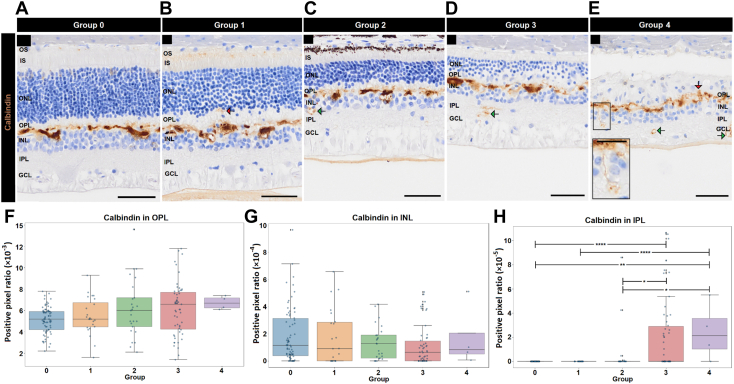
Calbindin labeling in relation to Outer Retina ratio. Representative image showing 3,3′-diaminobenzidine (DAB) staining of calbindin (brown) and 4′,6-diamidino-2-phenylindole (DAPI; blue) in parts (**A**) group 0 (control eye), (**B**) group 1, (**C**) group 2, (**D**) group 3, and (**E**) group 4. Red arrow in part (**B**) indicates calbindin into the OPL toward the ONL and green arrows in parts (**C–E**) show penetration of calbindin through the INL into the IPL. Neural sprouting is indicated in the black box insert. Graphs show quantification of calbindin in the (**F**) OPL, (**G**) INL, and (**H**) IPL for groups 0 (n = 75), 1 (n = 24) 2 (n = 25), 3 (n = 55), and 4 (n = 4). Scale bars are 50 μm. GCL = ganglion cell layer; INL = inner nuclear layer; IPL = inner plexiform layer; IS = inner segments; ONL = outer nuclear layer; OPL = outer plexiform layer; OS = outer segments. ∗*P* < 0.05; ∗∗*P* < 0.01; ∗∗∗∗*P* < 0.0001.

**Figure 8:**
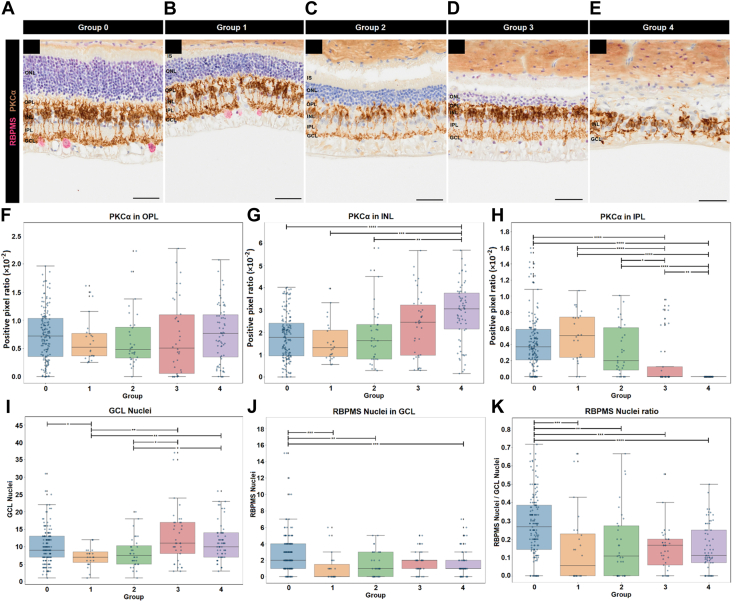
Protein kinase C alpha (PKCα) and RNA-binding protein with multiple splicing (RBPMS) labeling in relation to Outer Retina ratio. Representative image of PKCα (brown) and RBPMS (pink) in parts (**A**) group 0 (control eye), (**B**) group 1, (**C**) group 2, (**D**) group 3, and (**E**) group 4. Graphs show (**F**) quantification of PKCα in the OPL, (**G**) INL, and (**H**) IPL. Bottom graphs show (**I**) GCL nuclei count, (**J**) RBPMS stained nuclei, and (**K**) normalized RBPMS nuclei ratio. n = 154, 24, 32, 38, and 65 images for groups 0, 1, 2, 3, and 4, respectively. Scale bars are 50 μm. GCL = ganglion cell layer; INL = inner nuclear layer; IPL = inner plexiform layer; IS = inner segments; ONL = outer nuclear layer; OPL = outer plexiform layer. ∗*P* < 0.05; ∗∗*P* < 0.01; ∗∗∗*P* < 0.001; ∗∗∗∗*P* < 0.0001.

Next, we assessed calbindin, calretinin, and PKCα, which are markers known to label neurons of the INL that undergo degenerative changes secondary to photoreceptor loss. Calbindin (marker of horizontal and amacrine cell subpopulations) showed evidence of aberrant immunoreactivity in the OPL and IPL in all ATP groups (Fig 6A–E, red and green arrows). This, however, only reached significance in the IPL, with greater immunoreactivity in moderately to severely degenerated retinae (groups 3 and 4) compared with the control (group 0) or mildly degenerated retinae (groups 1 and 2; *P* < 0.05–0.0001; Fig 6H). This was possibly due to aberrant sprouting of neurites (Fig 6E insert). Calretinin (marker of amacrine and ganglion cell subtypes) showed no changes in immunoreactivity aside from a single significant decrease between group 0 (control) and group 3 in the OPL (*P* < 0.001; Dunn test following Kruskal–Wallis; Fig S7, available at www.ophthalmologyscience.org). Protein kinase C alpha (marker of rod bipolar cells) showed no significant change in immunoreactivity in the OPL (Fig 8A–F), but an increase in the INL, reaching significance between group 4 versus less degenerative groups (*P* < 0.01–0.0001; Dunn test following Kruskal–Wallis; Fig 8G). A complementary loss of PKCα immunoreactivity was observed in the IPL, reaching significance for the groups 3 and 4 versus groups 0 to 2 (*P* < 0.05–0.0001; Dunn test following Kruskal–Wallis; Fig 8H).

### GCL Alterations Relative to ORr

Ganglion cell layer nuclei counts gave inconsistent results between ORr groups with a significant decrease in GCL nuclei count between group 0 and 1 (*P* < 0.05; Dunn test following Kruskal–Wallis) but a significant increase between groups 1 and 2 versus groups 3 and 4 (*P* < 0.05–0.01; Dunn test following Kruskal–Wallis; Fig 8I). Further assessment of retinal ganglion cells via RNA-binding protein with multiple splicing (RBPMS) labeling (marker of medium and large DAPI-, deep red fluorescing agent-, NeuroTrace-, and NeuN-stained cells in the GCL)^27^ revealed a significant decrease in RBPMS immunoreactive nuclei between group 0 and all ATP groups, except group 3 (*P* < 0.01–0.001; Dunn test following Kruskal–Wallis; Fig 8J). Finally, normalizing RBPMS nuclei counts to total GCL nuclei counts gave a consistent decrease of 44% for all ATP ORr groups compared with control (*P* < 0.01–0.0001; Dunn test following Kruskal–Wallis; Fig 8K).

### Cortical Electrophysiology Relative to ORr

As a final analysis, we assessed the relationship between ORr as well as other inner retinal metrics in regions of images that were directly above a single electrode, and the relevant elicited cortical activity from monopolar electrical stimulation of that electrode,^18^ to determine if retinal histology metrics could account for the variability in cortical responses.

### Cortical Thresholds

**Figure 9:**
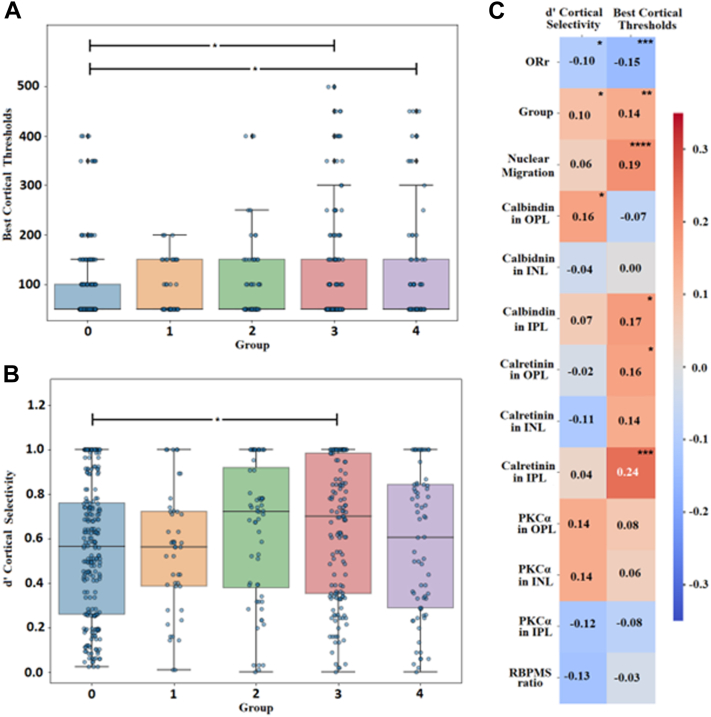
Correlations between retinal and cortical metrics in the adenosine triphosphate (ATP) model. (**A**) Relationship between best cortical thresholds and group. (**B**) Relationship between cortical selectivity and group. (**C**) Heatmap showing Spearman and Pearson correlation coefficients between best cortical threshold and d′ cortical selectivity against our measured inner retinal neural macromolecular markers, as well as nuclear migration, Outer Retina ratio (ORr), and groups. Calretinin in the inner plexiform layer (IPL) showed the highest level of significance and correlation to best cortical thresholds at 0.24 with a *P*-value < 0.0001, whereas the highest significant correlation found for d′ cortical selectivity was 0.16 with calbindin in the outer plexiform layer (OPL). Positive correlations are shown in red, negative in blue. d′ = d-prime; INL = inner nuclear layer; PKCα = protein kinase C alpha; RBPMS = RNA-binding protein with multiple splicing. ∗*P* < 0.05; ∗∗*P* < 0.01; ∗∗∗*P* < 0.001; ∗∗∗∗*P* < 0.0001.

Between ORr groups, there was a trend of increasing best cortical threshold (lowest value between ipsilateral and contralateral hemispheres) with significant difference in thresholds for the 2 most advanced categories, groups 3 and 4 to control group 0 (Fig 9A; group 0 = 82.43 ± 4.76 μA; group 1 = 88.64 ± 7.68 μA; group 2 = 98.25 ± 10.52 μA; group 3 = 117.25 ± 9.51 μA; and group 4 = 124.26 ± 13.97 μA; *P* < 0.05; Dunn test following Kruskal–Wallis). Correlation analyses supported this with a significant negative correlation between thresholds and ORr and a significant positive correlation between thresholds and ORr group (Fig 9C). However, both correlations were weak (r_ORr_ = –0.15, *P* < 0.001; r_Group_ = 0.14, *P* < 0.001). Weak, significant correlations between best cortical threshold and other retinal metrics were also found including presence of nuclear migration (*r* = 0.19, *P* < 0.0001), calbindin immunoreactivity in the IPL (*r* = 0.17, *P* < 0.05; Fig 9C), and calretinin immunoreactivity in the OPL (*r* = 0.16, *P* < 0.05; Fig 9C) or IPL (*r* = 0.24, *P* < 0.001; Fig 9C).

### Cortical Selectivity

The d′ cortical selectivity, which measures focal responsiveness of cortical activation from stimulation of each retinal electrode, was significantly increased by 11.9% in ATP-treated eyes versus control eyes (Fig 9B; d′_control_ = 0.54 ± 0.02; d′_ATP_ = 0.61 ± 0.02; *P* <0.01; Dunn test following Kruskal–Wallis). However, within ORr groups, a significant difference was only observed for group 3 versus control or group 0 (Fig 9B; group 0 = 0.54 ± 0.02; group 1 = 0.54 ± 0.04, *P* > 0.5; group 2 = 0.63 ± 0.04, *P* > 0.5; group 3 = 0.64 ± 0.03, *P* < 0.05; and group 4 = 0.57 ± 0.04, *P* > 0.5; Dunn test following Kruskal–Wallis). Correlation analyses revealed weak associations between d′ cortical selectivity and ORr value (*r* = –0.10, *P* < 0.05; Fig 9C) and ORr group (*r* = 0.10, *P* < 0.05; Fig 9C) as well as calbindin immunoreactivity of the OPL (*r* = 0.16, *P* < 0.05; Fig 9C).

## Discussion

This study showed that ORr can be an effective metric to quantify changes within degenerative retinae while accounting for heterogeneous photoreceptor loss. Outer Retina ratio categories correlated with existing metrics of photoreceptor loss and other features of degeneration and remodeling in the inner retina. However, it was limited in its ability to account for localized electrical stimulation efficacy in driving downstream cortical activity, pointing to a need to further develop metrics to assess relationships between specific retinal changes because of photoreceptor loss and treatment interventions such as a retinal prosthesis.

## ORr Highlights Intereye and Intraeye Variation in Photoreceptor Degeneration

Photoreceptor degeneration is known to exhibit both intereye and intraeye variation. Previous literature indicates that this variation is a significant issue, and methods that account for this and allow standardization between models are needed.^5^^,^^11^ However, most assessments of heterogeneity in these models have remained descriptive, with Follett et al^5^ recently describing the outcomes of each individual sample in their ground squirrel model to various pharmacological agents, highlighting the level of phenotypic variation. Our results indicate that ORr can account for intraeye variation, with horizontal cross-sections of the entire ATP-treated retina being successfully separated into 250 μm regions of no, mild, moderate and severe degeneration through ORr grouping. The distribution of ORr groups between our 4 animals further supported its utility in assessing intereye variation, with a larger proportion of group 1 versus group 3 regions in animals S1 and S2 versus S3 and S4, respectively, providing an objective method to indicate that the latter animals had more severe degeneration than the former, despite identical treatment conditions for all eyes. We acknowledge that although the measurement of outer retinal layers to quantify degeneration is not novel, the method used here, which assesses this metric using a high density, spatial approach, has not been considered before and is an effective method to allow researchers to categorize degeneration of individual animals receiving similar treatment. Other strengths of ORr include its ratio basis, which allows it to be robust to variations with eccentricity in layer thickness, and its calculation from retinal layers, which can be delineated in multiple imaging formats, meaning ORr may be applicable beyond microscopy images of histological retinal samples and could be applied to repeated, longitudinal, noninvasive *in vivo* clinical imaging using techniques such as OCT, as well as for histopathological analyses in other retinal degeneration animal models.

### ORr Highlights Disease Alterations That May Be Missed Using Pooled Analyses

Our analyses of outer and inner retinal macromolecular markers relative to ORr group demonstrated that significant variation in immunoreactivity existed within individual retinal samples and this may have been masked in previous pooled analyses. Specifically, we noted that reduced opsin immunoreactivity and retraction was present in all ORr groups of the ATP-treated retinae including group 1, which had not yet exhibited signs of photoreceptor loss. Several studies have indicated that alterations in opsin expression and localization occur in early-stage retinal degeneration and may be drivers of photoreceptor death.^28^, ^29^, ^30^ However, this report highlights such early alterations in the ATP-treated model and serves as a reminder not to assume that areas without outer retinal thinning in this model have been unaffected by the drug. We also found greater rhodopsin versus cone opsin changes in group 1 (and all subsequent groups), suggesting rod degeneration precedes cone degeneration in this model, which is supported by previous functional testing^8^ and mechanistic work indicating rod susceptibility to extracellular ATP mediated cell death through P2X_7_ activation or retinal pigment epithelium degeneration,^31^, ^32^, ^33^ and possible cone resistance to ATP via metabolic reliance on rods and Müller glia.^34^

Outer Retina ratio groups representing more severe stages of degeneration also highlighted changes associated with late-stage disease and inner retinal remodeling, coexisting within the sample set. Specifically, calbindin immunoreactivity was significantly increased in the IPL in groups 3 and 4 and neurite sprouting from the INL to the IPL was observed qualitatively in group 4, consistent with descriptions of microneuromas or sprouts from amacrine cells that migrate toward the GCL in stage 2 and midstage 3 retinal degeneration.^35^ In addition, the lack of systematic reduction and rather an increase GCL nuclei counts with some groups could also be explained by such inner retinal remodeling processes;^36^ however, we did observe a reduction in RBPMS labeled retinal ganglion cells. Meanwhile, PKCα immunoreactivity suggested evidence of retraction of rod bipolar cell processes with decreased immunoreactivity in the IPL and concurrent increased labeling in the INL in groups 3 and 4. Together, these findings suggest that the ATP feline model could be a useful model for the study of early versus late degeneration, as evidence of both stages seem to coexist in the model and can be objectively identified through ORr.

The implications of these findings in the latter ATP groups 3 and 4 suggest that moderate to late-stage remodeling can be predicted from these inner retinal changes, as these features were not as frequently observed in ATP groups 1 and 2. These findings have implications for device design, as stimulation paradigms and efficacy might differ because of changes that alter retinal circuitry and the function in moderate and late stages, though a clear connection remains elusive.

### ORr Alone Could Not Improve Assessment of Efficacy of a Vision-Restoration Intervention

Although intraeye and intereye variation in photoreceptor loss has been flagged as a major issue for the development and evaluation of electrically stimulated vision-restoration implants, stimulation efficacy from these devices has mostly been assessed as a function of postnatal day, with little inquiry into site-specific retinal alterations,^37^, ^38^, ^39^, ^40^ apart from our previous study relating glial responses to efficacy.^16^ When we assessed stimulation efficacy (either as best thresholds of neural activity or d′ cortical selectivity) with ORr in this study, we unfortunately found no strong direct relationships, but thresholds and d′ values, to some extent, were different when stimulating the more severely degenerated retinae (groups 3 and 4) versus control regions, further indicating some utility of ORr in this context. The weakness of the direct correlations might be due to the high degree of binocularity of the feline visual system^16^ and the limited cortical area sampled by the recording arrays, which did not cover the full extent of the visual field covered by the retinal area, adding to the variation in thresholds and cortical selectivity measures. Specifically, it has been observed in the feline that unilateral blinding might not produce cortical alterations, because of the intact visual representation delivered by the contralateral eye.^41^, ^42^, ^43^, ^44^

Notably, we found that the best thresholds of neural activity and d′ cortical selectivity were correlated with increases in nuclear migration and calbindin labeling in retinal plexiform layers, which suggests that these factors could be considered in directly influencing stimulation efficacy in driving cortical activity. As ORr can be grossly related to such features, it may potentially serve as a proxy for retinal alterations caused by photoreceptor loss that may alter/impede stimulation efficacy or information transmission from the retina to the cortex, which are otherwise difficult to estimate without invasive histological measurement. Additionally, it is likely that multiple overlapping metrics may be required to accurately relate proxy metrics to disease progression in nuanced ways. The implications of finding the right combination of remodeling metrics (particularly those that can be obtained clinically via noninvasive imaging or through analyses of retinal biopsies) that highly correlate with device performance would be a significant step forward in devising appropriate patient selection criteria for each intervention, to maximize the chances of success.

### Limitations

This study only examined a select number of well characterized retinal cell markers, which means the link between ORr and some cell populations remains to be established. Limitations in antibody availability and tissue processing incompatibility also meant that we performed both immunofluorescent staining and DAB histochemistry to visualize and quantify cell markers, meaning we were unable to perform more complex labeling and assess marker colocalization alongside ORr or cortical metrics. Future work could benefit from further exploration of relevant markers such as synaptic markers that may better elucidate axon and dendrite retraction or sprouting in more degenerative ORr groups. In addition, as ORr was a simple ratio of retinal thicknesses, it is possible that regions with similar ORr may have had differences in photoreceptor density or circuitry that were not detected, as they did not necessarily alter thickness. Reliance on outer retinal thickness for ORr calculation also caused issues in regions of severely degenerated retina where accurate delineation of OR was not possible. Future work in metric refinement could consider alternative methods of calculation, such as a ratio of layer labeling to the layer area, or alternate layer definitions, as well as validation across other models. Ultimately, ORr as a robust proxy for retinal remodeling, could become a useful aid to the interpretation of assessments of retinal degeneration made by pathologists, and could reduce variability across multiple assessments. Our technique could also provide a simple way for nonexperts to grade images, which can be helpful in large samples or to generate automated methods for classification.

### Conclusions

This study indicates that ORr is an eccentricity-agnostic proxy metric for photoreceptor loss that can be used to account for variation in photoreceptor loss and predicts certain canonical features of retinal remodeling. Outer Retina ratio and specific features of retinal degeneration only demonstrated weak correlations with cortical metrics, but this may be due to the multifactorial nature of the aberrancies that occur in the entire degenerate visual system and possibly limited alteration of the visual cortex due to the feline’s high binocularity.
