# Supplementary material for: Quantitative Markers of Neural Changes, Retinal Thickness, and Responses to Electrical Stimulation in Retinal Degeneration
Source: Ophthalmol Sci. 2026 Mar 27;6(6):101174. doi: 10.1016/j.xops.2026.101174 (PMC13186007; doi:10.1016/j.xops.2026.101174)
Supplement: Figure S3 [file mmc1.docx]

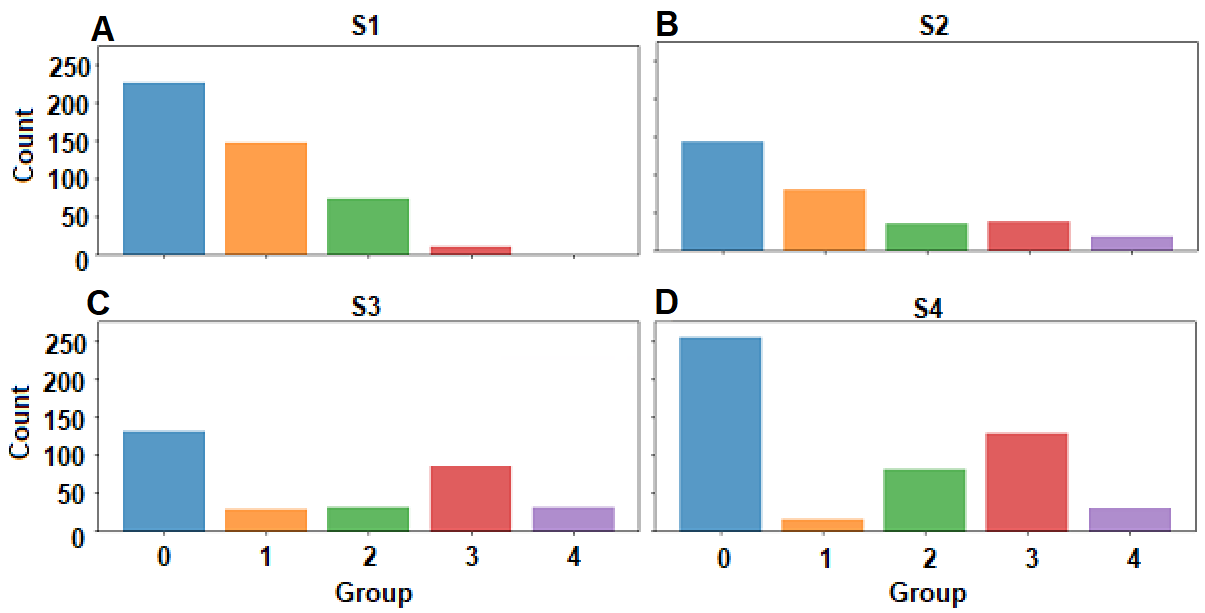


**Supplementary Figure S3**: Distribution of outer retina ratio groups per animal. Graphs **(A-D)** belong to subjects 1, 2, 3, and 4, respectively. Note that the distribution of outer retina ratio for the different subjects varied greatly, as feline 1 **(A)** showed little loss of outer retina thickness in relation to total retina, compared to feline 4 **(D)**, which had little seemingly healthy retinae in the ATP injected eye. Abbreviations: S1, Subject 1; S2, Subject 2; S3, Subject 3; S4, Subject 4.
