# Supplementary material for: Quantitative Markers of Neural Changes, Retinal Thickness, and Responses to Electrical Stimulation in Retinal Degeneration
Source: Ophthalmol Sci. 2026 Mar 27;6(6):101174. doi: 10.1016/j.xops.2026.101174 (PMC13186007; doi:10.1016/j.xops.2026.101174)
Supplement: Figure S7 [file mmc2.docx]

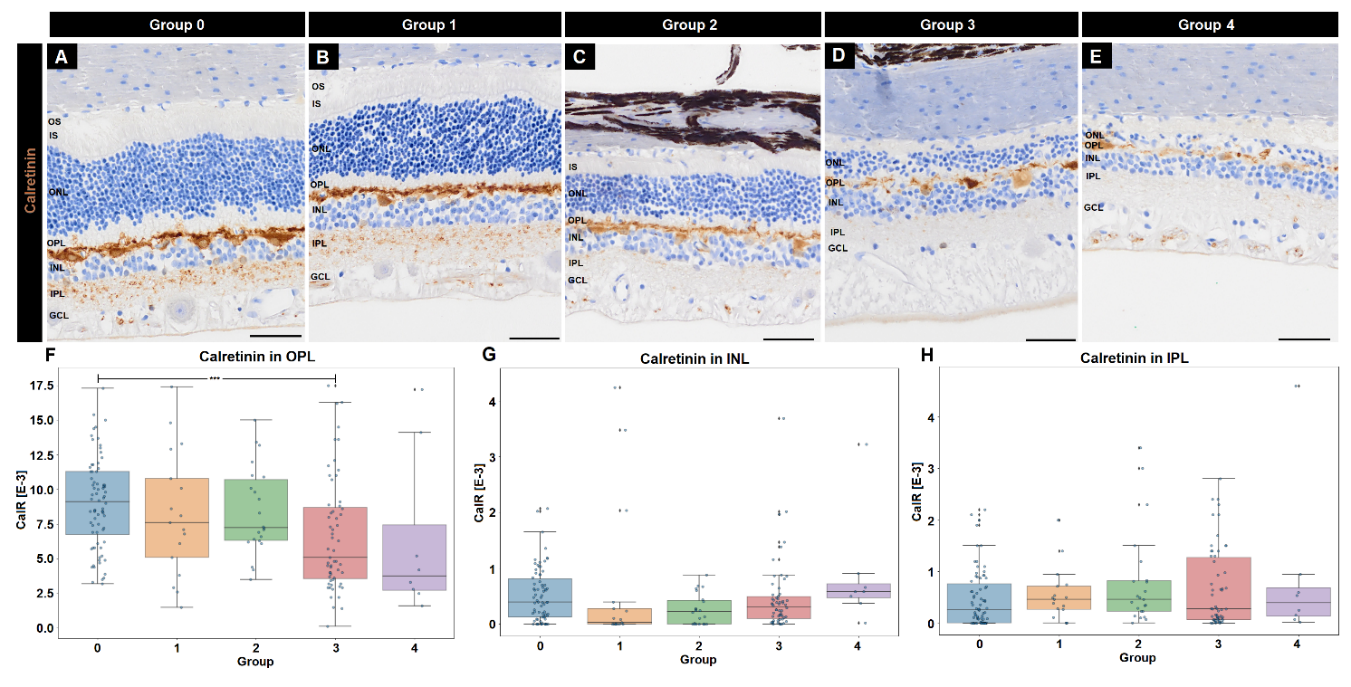


**Supplementary Figure S7**: Calretinin labelling in relation to ORr. Representative image of DAB staining of Calretinin (brown) and DAPI (blue) in **(A)** Group 0 (control eye) **(B)** Group 1, **(C)** Group 2, **(D)** Group 3 and **(E)** Group 4. Graphs show **(F)** quantification of Calbindin in the OPL, **(G)** INL and **(H)** IPL. Calretinin n = 76, 17, 22, 59, 8 images for groups 0, 1, 2, 3, and 4 respectively. Scale bars are 50 µm. Abbreviations same as in Figure 1. ***p < 0.001.
